# Effect of fibrate treatment on liver function tests in patients with the metabolic syndrome

**DOI:** 10.1186/2193-1801-3-14

**Published:** 2014-01-08

**Authors:** Nirav Gandhi, Richard Lenton, Mithun Bhartia, Ahmed Abbas, Jessie Raju, Sudarshan Ramachandran

**Affiliations:** College of Medical and Dental Sciences, University of Birmingham, Birmingham, UK; Heart of England Foundation Trust, Birmingham, UK; Diabetes and Endocrinology, Sandwell and West Birmingham Hospitals, Birmingham, UK; Southmead Hospital, North Bristol NHS Trust, Bristol, UK; Department of Clinical Biochemistry, Good Hope Hospital, Heart of England NHS Foundation Trust, Rectory Road, Sutton Coldfield, West Midlands B75 7RR UK

**Keywords:** Fibrates, GGT, ALT, ALP, PPARα, Metabolic syndrome, Statins

## Abstract

**Background:**

Fibrates are used especially in patients with hypertriglyceridaemia, a feature of the metabolic syndrome. Elevated LFTs are often observed in these patients perhaps related to fatty infiltration.

**Aim:**

We wished to study changes seen in LFTs (GGT, ALT and ALP) following fibrate therapy and then determine associated factors.

**Methods:**

This was a retrospective observational study in which data was collected from case notes of patients started on fibrates (n = 118, 2002–2008) in the lipid clinic at Good Hope Hospital and pre/post-fibrate lipid and LFT values were obtained. All biochemistry was performed on the Roche P-Unit using supplied reagents. Statistical analyses included t tests and regression analyses (factorised when quartiles were compared).

**Results:**

Of the study population 106 patients were on fenofibrate; the remaining on bezafibrate. Significant lowering of GGT (p < 0.0001), ALT (p = 0.0014) and ALP (p < 0.0001) levels were observed following fibrate treatment. Baseline lipid (cholesterol, triglycerides and HDL) concentrations, alcohol intake, length of treatment, gender, concurrent statin treatment and diabetes did not correlate with these changes in LFT in a multiple regression analysis. Higher pre-fibrate GGT (p < 0.0001), ALT (p < 0.0001) and ALP (p < 0.0001) concentrations were associated with larger decreases in each of these tests respectively with the highest 2 quartiles (GGT > 57 IU/l, ALT > 34 IU/l and ALP > 94 IU/l) significantly different to the lowest quartile. The above associations remained significant even when the regression analyses were corrected for changes in lipid values (which did not show an association).

**Conclusions:**

Fibrate treatment led to improvements in LFT, the greatest benefit seen in patients with higher baseline LFT values. It appears that baseline and changes in lipid values post fibrate treatment were not associated with change in LFT.

## Introduction

Fibrates are drugs that are often used to treat patients with the atherogenic lipoprotein phenotype, characterised by increased triglyceride levels and decreased levels of HDL-C (Staels et al., 1998a). This pattern of dyslipidaemia, central weight distribution, hypertension, and impaired fasting glucose/diabetes are characteristics of the metabolic syndrome. A decrease of 20–50% in serum triglyceride levels and an increase of 10–20% in HDL-C levels have been demonstrated in various studies following fibrate therapy (Brown et al., 1986; Farnier et al., 1994; Munoz et al., 1999; Poulter, 1999; Kiortsis et al., 2000). In contrast with the significant cardiovascular benefit consistently observed in statin trials (4S (The Scandinavian Simvastatin Survival Study, 1994), WOSCOPS (Shepherd et al., 1995), HPS (Heart Protection Study Collaborative Group, 2002), JUPITER (Ridker et al., 2008)), only VAHIT (Rubins et al., 1999) and HHS (Frick et al., 1987) of the fibrate trials have resulted in a significant reduction in primary outcome. However, subgroup analysis of all the fibrate trials (VAHIT (Rubins et al., 1999), HHS (Frick et al., 1987), ACCORD (ACCORD Study Group, 2010), BIP (The Bezafibrate Infarction Prevention (BIP) study, 2000) and FIELD (Keech et al., 2005)) and a meta-analysis by Jun et al (2010) have suggested that cardiovascular benefits appear to be maximal in subjects possessing characteristics of the metabolic syndrome.

Patients with the metabolic syndrome and uncontrolled dyslipidaemia of long duration are considered to be at greater risk of developing hepatic steatosis (Marchesini et al., 2001). A two or three hit hypothesis has been proposed: damage initially caused by fatty infiltration associated with insulin resistance and obesity; the “first hit”. This is followed by injury resulting from mechanisms linked to oxidative stress and impairment of cellular regeneration; the “second and third hits” (Dowman et al., 2010). The steatosis could progress through NASH (non-alcoholic steatohepatitis) and liver fibrosis to liver cirrhosis. The above spectrum of stages is collectively referred to as NAFLD (non-alcoholic fatty liver disease). Zelber-Sagi et al. demonstrated that 71% of patients with the metabolic syndrome had NAFLD (Zelber-Sagi et al., 2005). Individuals with NAFLD often have higher levels of insulin, glucose, TG, ALT, BMI and waist circumference. Thus, it is likely that a proportion of the patients with metabolic syndrome will indeed have some degree of NAFLD. Clinical features of NAFLD include hepatomegaly and moderate elevation in liver enzymes; including GGT and ALT, with steatosis often detected on ultrasound scanning (Franzini et al., 2012).

Management of NAFLD has focused on improving the characteristics classifying the metabolic syndrome; weight management, dyslipidaemia and glycaemia (Chalasani et al., 2012). As the pathogenesis of NAFLD is related to insulin resistance and oxidative stress, its therapeutic management currently includes insulin sensitisers, anti-oxidants as well as hepatoprotective drugs, which have shown promise in randomised controlled trials (Belfort et al., 2006; Harrison et al., 2003; Dufour et al., 2006). Probucol, an antioxidant, showed improved levels of ALT in comparison to a placebo over a six-month treatment period (Merat et al., 2003). Surgical intervention in patients who are morbidly obese, have shown to improve transaminases levels post-bariatric surgery (Mathurin et al., 2009). More research is required into these treatments to prove their efficacy.

Fibrate treatment has been associated with elevated transaminases. (AST and ALT) and this association appears to be dose related (Kobayashi et al., 2009). It is recommended that regular monitoring of liver function be carried out during fibrate therapy and the drug discontinued if levels persisted above ×3 the upper limit of the normal reference range (http://www.medicines.org.uk/emc/medicine/22425/SPC#UNDESIRABLE_EFFECTS). Fernandez-Miranda et al. carried out a pilot trial evaluating the effects of fenofibrate in 16 patients with NAFLD for 48 weeks (Fernandez-Miranda et al., 2008). Although significant improvement was observed in LFT and the metabolic syndrome, changes in liver histology was minimal. Interestingly no significant change in BMI was noted that could have been associated with the changes in LFT.

The dyslipidaemia clinics run at Good Hope Hospital (part of the Heart of England NHS Foundation Trust) have used fibrates in patients with severe hypertriglyceridaemia and those with the atherogenic lipoprotein phenotype, the dyslipidaemia seen in the metabolic syndrome. Many of these patients had elevated baseline LFT and regular monitoring was carried out in view of the association with elevation of transaminases. We wished to determine whether significant changes in LFT, such as observed in the study by Fernandez-Miranda (Fernandez-Miranda et al., 2008) took place following fibrate therapy and in the event of changes, establish associated predictive factors.

## Methods

This was a retrospective observational study. All patients commenced on fibrates between 2002 and 2008 in the lipid clinic at Good Hope Hospital, (Birmingham, United Kingdom) were included in the study. In all cases the decision to commence fibrate treatment was made on clinical grounds taking current evidence and individual lipid profile into consideration. Our cohort of 118 patients was identified from the electronic record database by using the keywords relevant to this study. All the patients fulfilled the metabolic syndrome diagnostic criteria (NCEP/ATPIII) (ATP3/NCEP definition (Expert Panel on Detection, Evaluation, and Treatment of High Blood Cholesterol in Adults), 2001). Table 1 demonstrates that 60 patients (52.6%) were already on statin treatment and there were no significant differences in baseline ALT and GGT as compared to those not on statins. There was however a small but significant difference in ALP levels in patients on statins (87.4 IU/L) and those not on statins (76.2 IU/L), p = 0.03. Increased ALP activity has been previously reported with statin treatment (Walter et al., 2013).

**Table 1:** **Characteristics of the patient group studied**

	N	%	Mean (SD)	Median	Range
**Age**	118		52.9 (10.3)	53.2	32.3–72.5
**Males**	76	68.5			
**Females**	35	31.5			
**Duration of treatment (years)**	110		0.5 (0.4)	0.3	0.02–2.1
**Diabetes**	31	28.2			
**On concurrent statin treatment**	60	52.6			
**Never smoked**	15	13.8			
**Ex-smokers**	77	70.6			
**Current smokers**	17	15.6			
**Alcohol (units/week)**					
0	16	16.8			
1–28	55	57.9			
>28	24	25.8			
**Pre-fibrate treatment**					
TC (mmol/l)	117		6.8 (2.6)	6.1	3.4–20.2
TG (mmol/l)	118		7.3 (7.8)	5.1	1.4–58.1
HDL-C (mmol/l)	98		1.1 (0.2)	1.1	0.7–1.9
Creatinine (μmol/l)	109		84.1 (16.8)	84	33–127
GGT (IU/l)	118		79.2 (59.8)	57.5	13–274
ALT (IU/l)	116		43.1 (33.5)	33.5	9–249
ALP (IU/l)	118		81.2 (24.2)	77.5	34–177
**Post-fibrate treatment**					
TC (mmol/l)	118		5.8 (1.4)	5.6	3–11.2
TG (mmol/l)	118		3.7 (3.8)	2.7	0.8–34.4
HDL-C (mmol/l)	115		1.2 (0.3)	1.1	0.6–2.5
Creatinine (μmol/l)	115		95.1 (20.0)	92	59–143
GGT (IU/l)	116		57.1 (43.6)	42	13–307
ALT (IU/l)	116		37.5 (24.5)	30	12–154
ALP (IU/l)	118		62.0 (19.9)	59	23–147

Data for the study was obtained from patient notes and electronic results documentation (Table 1; this also demonstrates the scale of missing data). It should be noted that all our patients received lifestyle management advice prior to drug treatment being considered. All patients had pre-fibrate treatment levels of TC, TG, HDL-C, creatinine, GGT, ALT and ALP measured. The post-treatment levels obtained were the most current results available (single determinants) or the results prior to introduction of another drug/discontinuation of the fibrate. As 88% of patients were on fenofibrate 160 mg we could not study the dose response phenomena on LFT change.

All biochemistry was performed on the Roche P-Unit using supplied reagents. The data was entered on a Microsoft Excel spreadsheet and transferred to the STATA (version 8.0 for Windows) statistical analysis software. Paired *t*-test was carried out to study changes in LFT (GGT, ALT and ALP) following fibrate treatment and differences between the cohorts stratified by different characteristics (e.g. patients on statins/not on statins). Separate linear regression analyses were then performed with changes in LFT (GGT, ALT and ALP) as the dependent variables and baseline characteristics and changes in lipids as the independent variables (model 1 of Tables 2, 3 and 4). It was possible that co-linearity existed between some of the above factors. Thus, independence was established by entering all the significant factors as independent variables in a multivariate regression analyses (model 2 of Tables 2, 3 and 4).

**Table 2:** **Association between baseline factors and change in GGT following fibrate treatment**

Model 1: linear regression analysis with change in GGT as the dependent variable and the following as independent variables in separate analyses				
	n	Co-efficient	95% C.I.	p-value
**Gender (male)**	116	−21.04	−37..03- –5.06	**0.010**
**Pre-TC**	115	−3.28	−6.18- –0.38	**0.027**
**Pre-TG**	116	−1.08	−2.03- –0.14	**0.025**
**Pre fibrate GGT**	116	−0.47	−0.56- –0.38	**<0.001**
**Pre fibrate ALT**	114	−0.28	−0.51- –0.05	**0.0180**
**Treatment duration**	117	19.7	1.35-38.0	0.036
**change in HDL-C**	98	−33.79	−57.19- –10.38	**0.005**
**Model 2: multiple regression analysis with change in GGT as the dependent variable and the following as independent variables in a single analysis.**				
	**n**	**Co-efficient**	**95% C.I.**	**p-value**
**Gender (male)**	96 (r^2^ = 0.52)	−5.47	−18.77-7.83	**0.42**
**Pre-TC**		0.48	−3.81-4.77	**0.83**
**Pre-TG**		−1.43	−3.77-0.91	**0.23**
**Pre fibrate GGT**		−0.44	−0.56- –0.31	**<0.001**
**Pre fibrate ALT**		0.07	−0.12-0.26	**0.48**
**Treatment duration**		12.5	1.86-26.9	**0.087**
**change in HDL-C**		−24.61	−55.42-6.22	**0.12**

Age, pre-fibrate HDL-C, pre-fibrate ALP, pre-fibrate creatinine, change in TC, change in TG were not significantly associated with changes observed in GGT following fibrate treatment.

**Table 3:** **Association between baseline factors and change in ALT following fibrate treatment**

Model 1: linear regression analysis with change in ALT as the dependent variable and the following as independent variables in separate analyses				
	n	Co-efficient	95% C.I.	p-value
**Pre fibrate GGT**	114	−0.1	−0.15- –0.05	**<0.001**
**Pre fibrate ALT**	114	−0.36	−0.43- –0.28	**<0.001**
**Model 2: multiple regression analysis with change in ALT as the dependent variable and the following as independent variables in a single analysis.**				
	**n**	**Co-efficient**	**95% C.I.**	**p-value**
**Pre fibrate GGT**	114 (r^2^ = 0.45)	−0.03	−0.08-0.012	**0.16**
**Pre fibrate ALT**		−0.33	−0.41- –0.25	**<0.001**

Age, gender, pre-fibrate lipids (TC, TG & HDL-C), pre-fibrate ALP, pre-fibrate creatinine, treatment duration and changes in lipids (TC, TG & HDL-C) were not significantly associated with changes observed in ALT following fibrate treatment.

**Table 4:** **Association between baseline factors and change in ALP following fibrate treatment**

Model 1: Linear regression analysis with change in ALP as the dependent variable and the following as independent variables in separate analyses				
	n	Co-efficient	95% C.I.	p-value
**Pre-fibrate GGT**	118	−0.02	−0.10- –0.002	**0.04**
**Pre-fibrate ALP**	118	−0.38	−0.48- –0.28	**<0.001**
**Treatment duration**	117	6.36	0.92-11.81	**0.022**
**Statin treatment**	118	6.63	0.75-12.5	**0.028**
**Model 2: Multiple regression analysis with change in ALP as the dependent variable and the following as independent variables in single analysis**				
	**n**	**Co-efficient**	**95% C.I.**	**p-value**
**Pre-fibrate GGT**	117 (r^2^ = 0.33)	0	−0.04-0.04	**0.96**
**Pre-fibrate ALP**		0.36	0.26-0.47	**<0.001**
**Treatment duration**		−4.56	−9.18-0.07	**0.054**
**Statin treatment**		2.00	−3.08-7.08	**0.44**

Age, gender, diabetes, pre-fibrate lipids (TC, TG & HDL-C), pre-fibrate ALT, pre-fibrate creatinine, changes in lipids (TC, TG & HDL-C) were not significantly associated with changes observed in ALP following fibrate treatment.

## Results

Most of our patients (106 of 118; 89.8%) were on fenofibrate, whilst the remainder was on bezafibrate. Table 1 shows the general characteristics of the cohort and values for all relevant biochemical markers both pre and post-fibrate treatment irrespective of gender, smoking status, alcohol intake, diabetes status and concurrent statin treatment. Significant changes were seen in TC, TG and HDL-C following fibrate treatment. These findings were anticipated as our patient group was a subset of the cohort previously reported regards the above analytes (Ramachandran et al., 2012; Abbas et al., 2012). All three LFT studied decreased (Table 1) with fibrate treatment with paired *t*-Test showing the changes to be significant; GGT (p < 0.0001), ALT (p = 0.0014) and ALP (p < 0.0001).

### Factors associated with changes in LFT following fibrate treatment

We now wished to determine factors associated with the changes observed above in separate regression analyses. The change in LFT (GGT, ALT and ALP) were dependent variables with age, gender, duration of fibrate treatment, pre-treatment lipids (TC, TG and HDL-C), changes in lipids (TC, TG and HDL-C), statin treatment and pre-treatment LFT (GGT, ALT and ALP) entered as independent variables.

#### Change in GGT levels

Male gender, pre-treatment TC, pre-treatment TG, pre-treatment GGT, pre-treatment ALT, treatment duration and change in HDL-C were all significantly associated with changes in GGT (Table 2: Model 1). Multiple regression analysis (Table 2: Model 2) showed that only pre-treatment GGT levels (p < 0.001) remained significant.

#### Change in ALT levels

Table 3: model 1 shows that pre-treatment GGT (p < 0.001) and pre-treatment ALT (p < 0.001) were associated with changes in ALT following fibrate treatment. Pre-treatment ALT levels remained significant (p < 0.001) when both factors were entered in a multiple regression model.

#### Change in ALP levels

Table 4: Model 1 shows that pre-treatment GGT (p = 0.04), pre-treatment ALP (p < 0.001), statin treatment (p = 0.028) and treatment duration (p = 0.022) were significantly associated with changes observed in ALP following fibrate treatment. Only pre-treatment ALP (p < 0.001) remained significant on multiple regression.

### Association between LFT changes following fibrate and pre-treatment LFT

We have demonstrated that improvements in GGT, ALT and ALP were associated with fibrate therapy. The best predictor of this improvement appears to be the pre-treatment level of that particular analyte (GGT, ALT and ALP). The coefficients obtained from the regression analyses suggested that patients with higher baseline concentrations were more likely to see greater decreases in the respective LFT following fibrate treatment.

**Figure 1:**
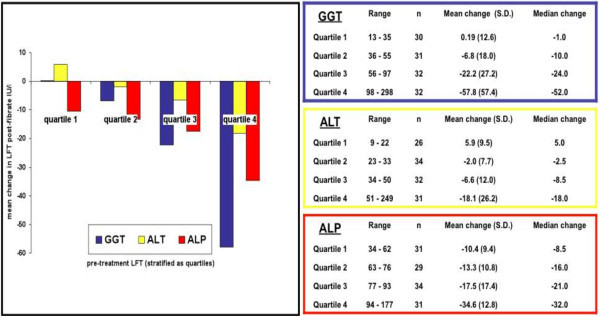
**Change in LFT following fibrate treatment pre-fibrate LFT stratified as quartiles.**

We wished to further characterise this observation. Patients were stratified into quartiles based on the pre-treatment GGT, ALT and ALP and the changes seen in these analytes presented in Figure 1. Details of the quartiles for each of the LFT (pre-treatment range and details of the change observed (mean and median) are presented in the figure. Regression analyses were performed with changes in GGT, ALT and ALP as dependent variables in separate analyses with pre-treatment GGT, ALT and ALP now factorised; in the previous regression analyses they were continuous variable. Quartile 1 was considered as the reference category and the other quartiles compared to this (Figure 1). The quartiles 3 and 4 (GGT ≥ 56 IU/l, ALT ≥ 34 IU/l and ALP ≥ 77 IU/l) were significantly different to the reference quartile 1 in each of the regression models. The above associations remained significant even when the regression analyses were corrected for changes in lipid values (which were not significantly associated themselves).

## Discussion

Our study suggested improvements in GGT, ALT and ALP following fibrate treatment. The decrease of each of these enzymes was greater in patients with higher baseline values. NAFLD/NASH is a common feature of the metabolic syndrome. As all our patients had dyslipidaemia characteristic of the metabolic syndrome it is reasonable to speculate that improvements in NAFLD/NASH may have contributed to improving LFT. However it should be noted that the precise relationship between abnormal LFTs and NASH outcomes requires further clarity. To be more certain of our hypothesis a liver biopsy where fat content can be directly measured would have to be carried out before and after treatment. This is not currently part of our routine clinical management. Other investigations such as ultrasound, CT/MRI scans although useful in confirming the presence of fat, have limitations (http://www.worldgastroenterology.org/assets/export/userfiles/2012_NASH%20and%20NAFLD_Final_long.pdf). Inadequate sensitivity and specificity together with an inability to quantify hepatic fat content restrict the value of these investigations in studies such as ours. Other hepatic pathology was excluded when clinically suspected or in patients whose LFT was greater than 5 times the ULN. Investigations here included further biochemistry/immunology as well as serology (viral hepatitis) and radiology.

The best predictors of LFT decrease appeared to be the baseline levels of that particular enzyme. Current views on the pathogenesis of NAFLD/NASH may provide an explanation. A two hit hypothesis was initially proposed with accumulation of hepatic fat, possibly associated with insulin resistance leading to inhibition of fatty acid oxidation considered the “first hit” (Day and James, 1998; Day, 2006). Factors that were thought to constitute the second hit include oxidative stress, mitochondrial abnormalities and hormonal imbalance (e.g. adiponectin and leptin levels). Since then the possibility of a “third hit”, factors impairing hepatocyte regeneration and proliferation has been suggested (Dowman et al., 2010; Jou et al., 2008).

Fibrates bind PPARα nuclear receptors and following dimerisation with RXR forms PPRE that in turn regulates gene transcription (genes regulated include ApoA1, ApoA2, ApoA5, LPL, ABCA1, ABCG1 and SRB1) (Auwerx et al., 1996). Increased mitochondrial fatty acid oxidation has been observed following PPARα activation in murine studies (Kerten et al., 1999). A functional PPARα appears to be a prerequisite for fatty acid catabolism and PPARα null mice show elevated free fatty acids (Aoyama et al., 1998). The liver produces VLDL continuously from endogenous synthesis, cytosolic TG stores, plasma lipoproteins and circulating free fatty acids. Thus, we speculate that fibrates may reduce hepatic fat, the “first hit” (Day, 2006). PPARα is also expressed in other cells/tissues; endothelium, smooth muscle, monocytes and macrophages (Chinetti et al., 1998; Inoue et al., 1998; Staels et al., 1998b). It may play a role in inhibition of inducible nitric oxide synthase gene transcription in macrophages thereby limiting inflammation (Paukkeri et al., 2007). Animal studies have also suggested this, although it must be stated that the effects of fibrates on murine peroxisomes varies from that on humans (Doull et al., 1999). This suggests that the effect of the “second hit” may also be limited (Jou et al., 2008).

Thus, fibrates may have a beneficial effect on both fatty acid oxidation and inflammation. Due to this we speculate that improvement in LFT associated with fibrates may be due to interrupting the pathogenesis of NAFLD/NASH at various points. In diseases of multifactorial aetiology a reduction in the risk posed by individual factors (e.g. changes in lipid levels following fibrate treatment) may not predict the changes observed in LFT. The presence of baseline NAFLD/NASH would be expected to be a better predictor as it would be associated with all the causative risk factors. This may be the reason that baseline LFT (which may be associated with NAFLD/NASH in our patients with the metabolic syndrome) was the best predictor of benefit following fibrates.

The above points raised are based on the assumption that the LFT elevation was associated with NAFLD/NASH. It is followed by a suggestion that the decrease in LFT levels was due to improvements in NAFLD/NASH. We accept that these may be considered steps too far. Regression to the mean is also a possibility. However, by making the point we would like to see similar studies in the future, but with confirmation and an estimate of hepatic fat content via non-invasive radiography. Corroboration of Fibroscan/Steatoscan results with biochemical markers of fibrosis would be interesting in a prospective cohort with a control group.

Very few clinical trials have examined the effects of fibrates on NAFLD (Nakajima, 2012). Laurin J et al. report no significant change in liver histology or enzymes (except for ALP) in 16 patients receiving clofibrate therapy for 12 months (Laurin et al., 1996). Basaranoglu M et al. administered gemfibrozil to 17 patients for 4 weeks and demonstrated a significant reduction in mean serum ALT, AST and GGT levels in comparison with the control group (Basaranoglu et al., 1999). The most recent clinical study in the literature examining the effects of fibrates on NAFLD was a pilot trial by Fernandes-Miranda in which 16 patients were administered fenofibrate for 48 weeks (Fernandez-Miranda et al., 2008). A significant decrease in abnormal LFT was noted compared to the control group although liver histology did not change significantly. Our study is the largest to date examining the effect of fibrates on LFT and also the first to demonstrate any association linked with this effect.

### Compliance with ethical requirements

All procedures followed were in accordance with the ethical standards of the responsible committee on human experimentation (institutional and national) and with the Helsinki Declaration of 1975, as revised in 2008. Our retrospectively audited data collection was from an electronic database of patients that attended our dyslipidaemia clinic and in whom any interventions were performed as part of their clinical treatment. Approval was obtained from the Heart of England NHS Foundation Trust.

### Randomised controlled trials

4S, Scandinavian Simvastatin Survival Study; WOSCOPS, West of Scotland Coronary Prevention Study; HPS, Heart Protection Study; JUPITER, Justification for the Use of Statins in Primary Prevention- An Intervention Trial Evaluating Rosuvastatin; VAHIT, Veterans Affairs High-Density Lipoprotein Intervention Trial; HHS, Helsinki Heart Study; ACCORD, Action to Control Cardiovascular Risk in Diabetes; BIP, Bezafibrate Infarction Prevention Study; FIELD, Fenofibrate Intervention and Event Lowering in Diabetes.

## Abbreviations

LFT: Liver function tests
GGT: Gamma-glutamyl transpeptidase
ALT: Alanine transaminase
ALP: Alkaline phosphatase
HDL-C: High density lipoprotein cholesterol
NAFLD: Non-alcoholic fatty liver disease
NASH: Non-alcoholic steatohepatitis
BMI: Body mass index
NCEP/ATPIII: National Cholesterol Education Program/Adult Treatment Panel III
PPARα: Peroxisome proliferator activated receptor alpha
RXR: Retinoid x receptor
PPRE: Peroxisome proliferator response element.
